# Supplementary material for: Dynamic analysis of circulating tumor DNA to predict the prognosis and monitor the treatment response of patients with metastatic triple-negative breast cancer: A prospective study
Source: eLife. 2023 Nov 6;12:e90198. doi: 10.7554/eLife.90198 (PMC10627511; doi:10.7554/eLife.90198)
Supplement: Supplementary file 1. — Footnotes: Listed in this file are 457 genes, known to be frequently mutated in tumors, and were designed to compose a targeted NGS panel in this study to capture targeted DNA segments. [file elife-90198-supp1.docx]

Supplementary File 1. The list of 457 genes detected in this study.

| ABL1 | BRCA1 | CDKN2C | ERBB3 | GABRA6 | IRF4 | MAPK8IP1 | NOTCH4 | POLE | RPA3 | TEK |
| --- | --- | --- | --- | --- | --- | --- | --- | --- | --- | --- |
| ACVR1B | BRCA2 | CEBPA | ERBB4 | GAGE1 | IRS2 | MCL1 | NPM1 | POLQ | RPL22 | TENT5C |
| ACVR2A | BRD4 | CHEK1 | ERCC2 | GALNT12 | ITK | MDM2 | NRAS | PPARG | RPL5 | TET1 |
| AJUBA | BRIP1 | CHEK2 | ERCC3 | GATA1 | JAK1 | MDM4 | NRG1 | PPM1D | RPTOR | TET2 |
| AKT1 | BTG1 | CIC | ERCC4 | GATA2 | JAK2 | MECOM | NSD1 | PPP2R1A | RUNX1 | TGFB1 |
| AKT2 | BTG2 | CREBBP | ERCC5 | GATA3 | JAK3 | MED12 | NSD2 | PPP2R2A | SDHA | TGFBR2 |
| AKT3 | BTK | CRIPAK | ERG | GATA4 | JUN | MEF2B | NSD3 | PRDM1 | SDHB | TIPARP |
| ALK | BTLA | CRKL | ERRFI1 | GATA6 | KDM5A | MEN1 | NT5C2 | PRF1 | SDHC | TLR4 |
| ALOX12B | EMSY | CSF1R | ESR1 | GID4 (C17orf39) | KDM5C | MERTK | NT5E | PRKAR1A | SDHD | TNF |
| AMER1 | CALR | CSF3R | ETV1 | GNA11 | KDM6A | MET | NTRK1 | PRKCI | SETBP1 | TNFAIP3 |
| APC | CARD11 | CTAG2 | EZH2 | GNA13 | KDR | MGMT | NTRK2 | PRX | SETD2 | TNFRSF14 |
| AR | CASP8 | CTCF | FANCA | GNAQ | KEAP1 | MITF | NTRK3 | PTCH1 | SF3B1 | TNFRSF18 |
| ARAF | CBFB | CTLA4 | FANCC | GNAS | KEL | MKNK1 | P2RY8 | PTEN | SGK1 | TNFRSF4 |
| ARFRP1 | CBL | CTNNA1 | FANCG | GREM1 | KIT | MLH1 | PALB2 | PTK6 | SH2D1A | TNFSF11 |
| ARHGAP35 | CCND1 | CTNNB1 | FANCL | GRM3 | KITLG | MLH3 | PRKN | PTPN11 | SIK1 | TNFSF14 |
| ARHGEF12 | CCND2 | CUL3 | FANCM | GSK3B | KLHL6 | MPL | PARP1 | PTPRD | SIN3A | TNFSF18 |
| ARID1A | CCND3 | CUL4A | FAS | H3F3A | KMT2A | MRE11 | PARP2 | PTPRK | SLAMF7 | TNFSF4 |
| ARID2 | CCNE1 | CXCR4 | FBXW7 | H3F3C | KMT2B | MSH2 | PARP3 | PTPRO | SMAD2 | TOP1 |
| ARID5B | CD160 | CYP17A1 | FGF10 | HAVCR2 | KMT2C | MSH3 | PAX5 | PTPRT | SMAD4 | TP53 |
| ASXL1 | CD22 | DAXX | FGF12 | HDAC1 | KMT2D | MSH6 | PBRM1 | QKI | SMARCA4 | TSC1 |
| ATM | CD244 | DDR1 | FGF14 | HDAC2 | KRAS | MST1R | PCBP1 | RAC1 | SMARCB1 | TSC2 |
| ATR | CD274 | DDR2 | FGF19 | HDAC3 | LAG3 | MTAP | PCNA | RAD21 | SMC1A | TSHR |
| ATRX | CD276 | DICER1 | FGF23 | HDAC6 | LCK | MTOR | PDCD1 | RAD50 | SMC3 | TSHZ2 |
| AURKA | CD28 | DIS3 | FGF3 | HGF | LEF1 | MUTYH | PDCD1LG2 | RAD51 | SMO | TSHZ3 |
| AURKB | CD38 | DNMT3A | FGF4 | HIST1H1C | LIFR | MYC | PDGFRA | RAD51B | SNCAIP | TYRO3 |
| AXIN1 | CD48 | DOT1L | FGF6 | HIST1H2BD | LIMK1 | MYCL | PDGFRB | RAD51C | SOCS1 | U2AF1 |
| AXIN2 | CD69 | EED | FGFR1 | HNF1A | LRRK2 | MYCN | PDK1 | RAD51D | SOX17 | USP9X |
| AXL | CD70 | EGFR | FGFR2 | HRAS | LTK | MYD88 | PHF6 | RAD52 | SOX2 | VEGFA |
| B2M | CD79A | EGR3 | FGFR3 | HSD3B1 | LYN | NAV3 | PHOX2B | RAD54L | SOX9 | VEGFB |
| B4GALT3 | CD79B | EIF4A2 | FGFR4 | ICOS | MAF | NBN | PIGF | RAF1 | SPATA2 | VEZF1 |
| BAGE | CD80 | ELF3 | FH | ICOSLG | MAGEA1 | NCOA4 | PIK3C2B | RARA | SPEN | VHL |
| BAP1 | CD86 | EOMES | FLCN | ID3 | MAGEA12 | NCOR1 | PIK3C2G | RB1 | SPOP | VTCN1 |
| BARD1 | CDC73 | EP300 | FLT1 | IDH1 | MAGEA3 | NEK11 | PIK3CA | RBM10 | SRC | WT1 |
| BCL2 | CDH1 | EPCAM | FLT3 | IDH2 | MAGEA4 | NF1 | PIK3CB | RECQL | STAG2 | XPO1 |
| BCL2L1 | CDK12 | EPHA1 | FLT4 | IGF1R | MAGEC2 | NF2 | PIK3CD | RECQL4 | STAT3 | XRCC2 |
| BCL2L2 | CDK4 | EPHA2 | FOXA1 | IGF2 | MAP2K1 | NFE2L2 | PIK3CG | REL | STK11 | ZNF217 |
| BCL6 | CDK6 | EPHA3 | FOXA2 | IKBKE | MAP2K2 | NFE2L3 | PIK3R1 | RET | SUFU | ZNF703 |
| BCOR | CDK8 | EPHB1 | FOXL2 | IKZF1 | MAP2K4 | NFKBIA | PIK3R2 | RICTOR | SYK |  |
| BCORL1 | CDKN1A | EPHB4 | FOXO3 | IL7R | MAP3K1 | NKX2-1 | PIM1 | RNF43 | TAF1 |  |
| BLM | CDKN1B | EPHB6 | FOXP1 | INPP4B | MAP3K13 | NOTCH1 | PMS1 | ROS1 | TAS2R38 |  |
| BMPR1A | CDKN2A | EPPK1 | FRK | INSR | MAPK1 | NOTCH2 | PMS2 | RPA1 | TBL1XR1 |  |
| BRAF | CDKN2B | ERBB2 | FUBP1 | IRF2 | MAPK11 | NOTCH3 | POLD1 | RPA2 | TBX3 |  |
